# Association between sociodemographic and injury factors in community reintegration following spinal cord injury: An analysis of two datasets

**DOI:** 10.4102/sajp.v82i1.2326

**Published:** 2026-03-26

**Authors:** Joyce Mothabeng, Chiedozie Eleje, Martins Nweke

**Affiliations:** 1 Department of Physiotherapy, Faculty of Health Sciences, University of Pretoria, Tshwane, South Africa; 2 Department of Physiotherapy, Faculty of Health Sciences, David UmahI Federal University of Health Sciences, Uburu, Nigeria

**Keywords:** spinal cord injury, community reintegration, sociodemographic factors, injury factors, Africa, cross-sectional studies, rehabilitation, secondary data analysis

## Abstract

**Background:**

Community reintegration after spinal cord injury (SCI) is influenced by personal and injury-related factors. However, data from two South African studies examining these associations have not been jointly analysed.

**Objectives:**

Our study aimed to examine the association between sociodemographic and injury-related factors and community reintegration among people with spinal cord injury (PWSCI).

**Method:**

Secondary data analysis was conducted using two cross-sectional South African datasets comprising 182 PWSCI. The datasets met the predefined homogeneity criteria and were analysed separately and as a pooled dataset. Community reintegration was measured using the Reintegration to Normal Living Index (RNLI). Descriptive statistics were used to summarise participant characteristics, while Chi-square and Fisher’s exact tests were applied to examine associations between explanatory variables and community reintegration outcomes.

**Results:**

In the pooled analysis, better community reintegration was significantly associated with younger age (< 55 years), female sex and employment (*p* < 0.05). Participants with thoracic and lumbar injuries showed higher levels of reintegration than those with cervical injuries although this association was not statistically significant. Injury severity and aetiology were not significantly associated with community reintegration.

**Conclusion:**

Younger age, female sex and favourable social circumstances, particularly employment, are associated with better community reintegration among PWSCI. These findings highlight the importance of considering the sociodemographic context and injury characteristics when planning rehabilitation and community-based support interventions.

**Clinical implications:**

Early identification of older and unemployed PWSCI may help clinicians target individuals at a higher risk of poor community reintegration and guide tailored rehabilitation strategies.

## Introduction

Community reintegration following spinal cord injury (SCI) is a multidimensional process influenced by social participation, environmental access and individual capacity (Akter et al., 2019; Craig et al. 2015; Dwyer & Mulligan 2015; Liu et al. 2023; Reinhardt et al. 2016). While numerous studies have examined the psychosocial and environmental determinants of reintegration, less attention has been paid to how sociodemographic and injury-related characteristics shape reintegration outcomes, particularly in low- and middle-income settings (Barclay et al. 2021; Quadri et al. 2020).

In sub-Saharan Africa, people with spinal cord injuries (PWSCI) often face compounded barriers related to unemployment, inaccessible built environments, limited psychological support and weak social systems (Atobatele et al. 2018; Kanyoni et al. 2024; Lijodi et al. 2025; Serres-Lafontaine et al. 2023). Studies in South Africa have documented these challenges, highlighting the influence of factors such as self-efficacy, access to assistive devices, outdoor mobility barriers and social support on reintegration outcomes (Buys et al. 2022; Nizeyimana, Phillips & Joseph 2024; Van der Westhuizen et al. 2017). However, existing analyses have largely focused on isolated outcomes, such as physical fitness, health behaviours and quality of life, without explicitly examining the association between sociodemographic and injury-related factors and community reintegration (Mashola & Mothabeng 2019; Van der Westhuizen et al. 2017).

Two South African cross-sectional studies independently collected detailed sociodemographic, injury-related and community reintegration data using the Reintegration to Normal Living Index (RNLI) (Mashola 2022; Van der Westhuizen 2016). Although both studies gathered relevant variables, the associations between sociodemographic factors, injury factors and community reintegration were not explored in the original analysis. Secondary data analysis provides an opportunity to maximise the scientific value of existing datasets by addressing unanswered research questions while reducing participant burden and optimising the use of limited research resources (Wickham 2019).

Therefore, our study aimed to examine the association between sociodemographic and injury-related factors and community reintegration among PWSCI using a secondary analysis of two comparable South African datasets.

### Objective

Our study aimed to examine the association between sociodemographic and injury factors and community reintegration in PWSCI in two separate settings.

## Research methods and design

### Conceptual framework

According to Nweke et al. (2025), when exploring exposure-outcome associations using multiple datasets from dissimilar settings, it is important to ensure that such datasets are similar in most other characteristics. Failure to ensure dataset similarity introduces systematic biases that can compromise both the internal and external validity (Grimes & Schulz 2002). In exposure-outcome associations, biases can result from confounding variables, selection methods and measurement errors that can alter a causal relationship (Grimes & Schulz 2002). In other words, the relationship between exposure and outcome may differ significantly if repeated several times using different selection and measurement methods. Hence, to ensure that the impact of exposure on the outcome is the object of exploration, efforts should be made to control potential biases in the secondary data analysis (SDA) of multiple datasets by ensuring that only datasets that are similar in important ways are merged (Nweke et al. 2025). As a rule of thumb, our study sets should be similar in four of the six study characteristics: study population, age, sex, sampling technique, study design and outcome measures (Nweke et al. 2025).

#### Dataset similarity and comparability

Dataset comparability was assessed using predefined homogeneity criteria based on our study design, population characteristics, outcome measurements and sampling approaches (Nweke et al. 2025). The two primary studies were cross-sectional, recruited adult PWSCI discharged from rehabilitation centres in Gauteng province and used the RNLI to measure community reintegration (Table 1). Both employed non-probability convenience sampling and collected data using interviewer-administered questionnaires. Following the guidelines for secondary analyses of multiple datasets, similarity was evaluated across six characteristics: study population, age range, sex distribution, sampling technique, study design and outcome measures. The datasets were aligned on at least four of these six characteristics, which were considered sufficient to permit pooled analysis while minimising heterogeneity. Table 1 presents a narrative comparison of these characteristics of the two groups. Formal statistical equivalence testing was not conducted because the datasets were not originally designed for equivalence assessment and differed in terms of sample size and recruitment scope. Triangulation was used to compare the findings of the individual and pooled analyses to assess the consistency of the observed associations.

**TABLE 1:** Narrative comparison of study characteristics between study one and study two.

Study author	Age range (years)	Female (%)	Sampling technique	Study setting	Study population	Study design	Outcome measure for community reintegration
Mashola (2022)	18−50+	33	Convenience 122	Gauteng, Mpumalanga, Limpopo	People with spinal cord injury (PWSCI)	Cross-sectional	RNLI
Van der Westhuizen (2016)	18−60+	16.67	Convenience 60	Tshwane South Africa	Adults with spinal cord injury (manual wheelchair users)	Cross-sectional	RNLI

Note: Please see full reference list of this article: Mothabeng, J., Eleje, C. & Nweke, M., 2026, ‘Association between sociodemographic and injury factors in community reintegration following spinal cord injury: An analysis of two datasets’, *South African Journal of Physiotherapy* 82(1), a2326. https://doi.org/10.4102/sajp.v82i1.2326 for more information.

RNLI, reintegration to normal living index.

### Design

Our study was an SDA of two primary cross-sectional studies conducted by Van der Westhuizen (2016) and Mashola (2022). The purpose of this SDA was to investigate the association between sociodemographic factors, injury factors and community reintegration among PWSCI. This was carried out by analysing the primary research data collected by Van der Westhuizen (2016) and Mashola (2022), involving variables such as sociodemographic information, injury factors and community reintegration in PWSCI. Our study was structured using the STROSA (Standardised Reporting of Secondary Data Analyses) checklist (Swart et al. 2016).

Setting: The SDA was conducted at the Department of Physiotherapy, University of Pretoria in South Africa. The two original research studies involved PWSCI discharged from rehabilitation centres located in Gauteng, with individuals residing in the provinces of Gauteng, Mpumalanga, Limpopo and North West.

### Study population

Our study population consisted of PWSCI discharged from rehabilitation centres located in Gauteng, but during data collection, the individuals lived in Gauteng, Mpumalanga, Limpopo and North West provinces after discharge. These participants were capable of providing informed consent and were able to respond to the RNLI questionnaire.

### Sampling

The original study used a non-probability convenience sample for the sample selection. However, in this SDA, total population sampling was employed, which involved all eligible records from the dataset. The sample consisted of 182 participants drawn from two primary studies: 60 from Van der Westhuizen (2016), the first study, and 122 from Mashola (2022), the second study. Patients were excluded if they did not meet the inclusion criteria, which Van der Westhuizen (2016) included being between 18 years and 70 years old, having a medical diagnosis of SCI, using a manual wheelchair, being able to push the wheelchair independently and having lived in the community for more than six months. In Mashola (2022), individuals younger than 18 years were excluded from the study. Only dataset records with complete data on sociodemographic factors, injury factors and community reintegration scores on the RNLI were selected for this SDA.

### Variables

In the SDA, the dependent variable was community reintegration. The exposure variables included sociodemographic characteristics, namely age, sex, marital status and employment status. The injury-related exposure variables were injury level, severity and type. Exposure variables (e.g. level of schooling and American Spinal Injury Classification [ASIA]) reported in only one dataset were excluded.

### Data collection

The datasets were derived from the original studies by Van der Westhuizen (2016) and Mashola (2022) and accessed from the dataset custodian at the Department of Physiotherapy, University of Pretoria. Primary data were collected using comprehensive and methodological processes, including standardised techniques and instruments. Community reintegration was assessed using the RNLI scale, whereas demographic and injury data were captured using sociodemographic and injury profiles (SDIP). Both the SDIP and RNLI were administered verbally to the participants. Variables (e.g., level of schooling and ASIA) reported in only one dataset were excluded. All data points were included, and community reintegration scores were reported.

Data collection began after the Faculty of Health Sciences Research Ethics Committee granted ethical approval. The Physiotherapy Department of the University of Pretoria granted access to the original dataset. The relevant variables were extracted from the original Excel spreadsheet to address the research question, and thereafter, a total of 182 records were reviewed for completeness. The extracted data were then organised in a new Excel spreadsheet, cleaned and recorded before analysis.

Missing data of relevant variables common to both datasets were identified and harmonised before data merging. Variables available in only one dataset were excluded to ensure consistency across analyses. Records were included only if complete data on sociodemographic variables, injury characteristics and RNLI scores were available. Consequently, a complete case analysis was performed. This approach was selected to maintain analytical consistency and avoid introducing bias through the imputation of secondary data that were not originally designed for pooled analysis.

### Data analysis

All variables of interest were categorical variables. Descriptive statistics were used to summarise the participant characteristics. Chi-square tests were used to examine the associations between sociodemographic and injury variables and community reintegration categories. Fisher’s exact test was used when the expected cell counts were low. Effect size was estimated using Cramér’s *V*. Multivariable modelling and data weighting were not performed because of the limitations inherent in the secondary datasets, including restricted variable availability and differences in sampling frames. Consequently, potential confounding factors could not be fully adjusted for, which is acknowledged as a limitation of our study. Analyses were performed using the statsmodels library (Seabold & Perktold 2010).

### Ethical considerations

Ethical clearance to conduct our study was obtained from the University of Pretoria Faculty of Health Sciences Research Ethics Committee at the University of Pretoria (No. 684/2024). The Physiotherapy Department at the University of Pretoria, the data custodian, granted permission to use these data sets.

All datasets used in our study were fully anonymised before being made available to authors. The datasets contained no direct personal identifiers (such as names, identity numbers or contact details), and indirect identifiers were removed or aggregated by the original data custodians in accordance with ethical and legal requirements. Consequently, individual participants could not be identified at any stage of analysis. Access to the datasets was granted under formal data-sharing agreements that limited their use to approved research objectives. These agreements prohibit the redistribution of raw data and restrict public sharing of aggregated results. Consequently, these datasets cannot be made openly available. However, requests for access may be directed to the original data custodians, subject to governance and approval processes.

## Results

### Comparison of participant characteristics across the two primary studies

This secondary data analysis included two primary cross-sectional studies: Van der Westhuizen (2016) (*n* = 60) and Mashola (2022) (*n* = 122). The age distribution of the participants did not differ significantly between the two studies (*χ*^2^ = 0.43, *p* = 0.515). However, significant differences were observed in the sex distribution, employment status and injury level (Table 2).

**TABLE 2:** Sociodemographic and injury characteristics of participants by dataset and pooled sample.

Variable	Category	Percentage	Dataset 1 Van der Westhuizen (2016) (*N* = 60)		Dataset 2 Mashola (2022) (*N* = 122)		*χ* ^2^	*df*	*p*-value
			*n*	%	*n*	%			
Age (years)	< 55	-	55	91.7	108	88.5	0.43	1	0.515
≥ 55	5	8.3	14	11.5	19	10.4	-	-	-
Gender	Male	-	50	83.3	83	68.0	4.79	1	0.029
Female	10	16.7	39	32.0	49	26.9	-	-	-
Employment status	Employed	-	48	80.0	44	36.1	31.06	1	< 0.001
Unemployed	12	20.0	78	63.9	90	49.5	-	-	-
Marital status	Married or cohabiting	-	26	43.3	65	53.3	1.59	1	0.207
Unmarried	34	56.7	57	46.7	91	50.0	-	-	-
Injury level	Cervical	-	22	36.7	0	0.0	52.99	2	< 0.001
Thoracic	31	51.7	111	91.0	142	78.0	-	-	-
Lumbar	7	11.6	11	9.0	18	9.9	-	-	-
Injury severity	Complete	-	46	76.7	93	76.2	1	0.948	-
Incomplete	14	23.3	29	23.8	43	23.6	-	-	-
Type of injury	Traumatic	-	55	91.7	104	85.2	1.5	1	0.22
Non-traumatic	5	8.3	18	14.8	23	12.6	-	-	-
Community reintegration (RNLI)	Severe	-	1	1.7	4	3.3	105.61	3	< 0.001
Moderate	4	6.7	94	77.0	98	53.8	-	-	-
Mild	25	41.6	24	19.7	49	26.9	-	-	-
Full	30	50.0	0	0.0	30	16.5	-	-	-

*Source*: Van der Westhuizen, L., 2016, ‘Correlation between physical activity and community participation in individuals with spinal cord injury’, MPhysT dissertation, University of Pretoria; Mashola, K.M., 2022, ‘The presence of pain and its impact on functioning and disability in manual wheelchair users with spinal cord injury: A framework for self-management’, PhD thesis, University of Pretoria, Pretoria

RNLI, reintegration to normal living index; *df*, degrees of freedom; χ^2^, Chi-squared.

Mashola (2022) included a higher proportion of male participants (83%) than Van der Westhuizen (2016) (68%) (*χ*^2^ = 4.79, *p* = 0.029). Employment status also differed markedly between studies, with a substantially higher proportion of unemployed participants in Mashola (2022) (64%) than in Van der Westhuizen (2016) (20%) (*χ*^2^ = 31.06, *p* < 0.001). Marital status did not differ significantly between the two datasets (*χ*^2^ = 1.59, *p* = 0.207) (Table 2).

With respect to injury characteristics, the distribution of injury levels differed significantly between studies (*χ*^2^ = 52.99, *p* < 0.001), with Mashola (2022) dominated by thoracic injuries and no cervical injuries, whereas Van der Westhuizen (2016) included cervical, thoracic and lumbar injuries. Injury severity and trauma type did not differ significantly between the two datasets (*p* > 0.05) (Table 2).

### Associations between sociodemographic and injury factors and community reintegration in the individual datasets

In separate analyses for each dataset, age was consistently and positively associated with reintegration. In Mashola (2022), younger participants were more likely to report higher levels of reintegration (*p* < 0.001), a pattern also observed by Van der Westhuizen (2016) (*p* = 0.009) (see Online Appendix 1 – Table 1-A1 and Table 2-A1). Gender was significantly associated with community reintegration in Van der Westhuizen’s study (2016), with female participants being more likely to report moderate reintegration than male participants (*p* = 0.019). This association was not observed by Mashola (2022) (*p* = 0.514) (Table 3). No statistically significant associations were identified between community reintegration and employment status, marital status, injury level, injury severity or trauma type in either dataset when analysed independently (all *p* > 0.05) (Online Appendix 1 – Table 1-A1 and Table 2-A1). These findings suggest that when considered separately, individual studies have limited power to detect associations beyond age and sex in one dataset (Gupta et al. 2019).

**TABLE 3:** Association between sociodemographic and injury factors and community reintegration in the pooled dataset.

Variable	Test	*χ* ^2^	*df*	*p*-value	Cramér’s *V*	Direction (highest standardised residuals)
Age (years) (< 55 vs ≥ 55)	Chi-square	16.44	2	< 0.001	0.292	Full reintegration more frequent among < 55
Gender (male vs female)	Chi-square	11.84	2	0.003	0.248	Moderate reintegration more frequent among females
Employment (employed vs unemployed)	Chi-square	7	2	0.030	0.190	Full reintegration more frequent among employed
Marital status (married/cohab vs unmarried)	Chi-square	0.24	2	0.887	0.035	No meaningful difference
Injury level (cervical/thoracic/lumbar)	Fisher’s exact	-	-	0.099	0.139	Higher reintegration tendency in thoracic/lumbar
Injury severity (complete vs incomplete)	Fisher’s exact	-	-	0.118	0.150	No consistent pattern
Type of injury (traumatic vs non-traumatic)	Chi-square	2.92	2	0.232	0.123	No consistent pattern

*Source*: Van der Westhuizen, L., 2016, ‘Correlation between physical activity and community participation in individuals with spinal cord injury’, MPhysT dissertation, University of Pretoria; Mashola, K.M., 2022, ‘The presence of pain and its impact on functioning and disability in manual wheelchair users with spinal cord injury: A framework for self-management’, PhD thesis, University of Pretoria, Pretoria

χ^2^, Chi-squared; *df*, degrees of freedom; vs, versus.

### Association between sociodemographic and injury factors and community reintegration in the pooled analysis

In the pooled analysis, several factors were found to be associated with community reintegration.

Sex was also significantly associated with community reintegration (*χ*^2^ = 11.84, *p* = 0.003), with female participants more frequently reporting moderate levels of reintegration than male participants. This association demonstrated a small-to-moderate effect size (Cramér’s *V* = 0.248). Employment status was significantly associated with community reintegration (*χ*^2^ = 7.00, *p* = 0.030). Employed participants were more likely to achieve full community reintegration than unemployed participants. The strength of this association was small (Cramér’s *V* = 0.190). In contrast, marital status was not significantly associated with community reintegration in the pooled dataset (*χ*^2^ = 0.24, *p* = 0.887), indicating a negligible effect (Cramér’s *V* = 0.035) (Table 3). With respect to injury-related factors, the level of injury did not reach statistical significance in the pooled analysis (Fisher’s exact test, *p* = 0.099) although participants with thoracic and lumbar injuries tended to report higher reintegration levels than those with cervical injuries. Injury severity and trauma type were not significantly associated with community reintegration (*p* = 0.118 and *p* = 0.150, respectively), and the effect sizes of these associations were small (Table 3).

### Triangulation of findings across individual and pooled analyses: A comparison of the results across the individual datasets and pooled analysis revealed consistent evidence

Injury-related factors did not demonstrate consistent or robust associations with community reintegration across the analyses (Table 3; Online Appendix 1 – Table 1-A1 and Table 2-A1). Therefore, the triangulation process supports the interpretation that sociodemographic characteristics, particularly age, gender and employment status, play a more prominent role in shaping community reintegration outcomes among people with SCI than injury characteristics.

## Discussion

Our study examined the association between sociodemographic and injury-related factors and community reintegration among people with spinal cord injury (PWSCI) using a secondary analysis of two South African datasets. Overall, the findings suggest that sociodemographic characteristics, particularly age, sex and employment status, are more consistently associated with community reintegration outcomes than injury-related factors. Importantly, these findings reflect associations rather than causal relationships and should be interpreted within the constraints of our study design.

Age demonstrated a consistent positive association with community reintegration across both individual and pooled analyses, with younger participants more likely to achieve higher levels of reintegration. This pattern aligns with the prior literature, indicating that younger participants may have greater physical resilience, social mobility and adaptability following SCI, as well as broader opportunities for employment and social participation (Buys et al. 2022; Craig et al. 2015). In the South African context, younger PWSCI may experience fewer age-related comorbidities and face fewer structural barriers to labour market participation, which may indirectly support reintegration.

Sex was associated with community reintegration in the pooled analysis and in one of the primary datasets. Female participants were more frequently represented in the moderate reintegration category. This finding should be interpreted cautiously, as gender differences in reintegration are complex and context-dependent (Pazzi et al. 2021). In South Africa, gender roles, caregiving networks and access to informal social support may shape men’s and women’s reintegration experiences differently. For example, women with disabilities may receive stronger family-based support, whereas men may experience greater social pressure related to employment and independence, which could influence perceived reintegration (Buys et al. 2022; Pazzi et al. 2021; Reinhardt et al. 2016).

Employment status emerged as a significant factor only in the pooled analysis, suggesting that an increased sample size enhanced the ability to detect associations. Employment is associated with higher levels of community reintegration, consistent with existing evidence that work facilitates social interaction, financial independence and a sense of purpose following SCI (Buys et al. 2022; Nizeyimana et al. 2024). In settings where unemployment rates are high and disability-related workplace accommodations remain limited (Morwane et al. 2021), employment may serve as a proxy for broader social inclusion and access to enabling environments rather than acting as a direct determinant of reintegration.

In contrast, marital status was not significantly associated with community reintegration in the pooled analysis, despite its frequent inclusion in rehabilitation research. This finding suggests that marital status alone may be an insufficient indicator of social support. Informal caregiving structures, extended family networks and community-based support may play a more meaningful role than formal marital status in shaping reintegration outcomes among individuals with spinal cord injuries in South Africa (Atobatele et al. 2018; Buys et al. 2022; Reinhardt et al. 2016).

Injury-related factors showed weaker and less consistent associations with community reintegration than other factors. Although participants with thoracic and lumbar injuries tended to report higher reintegration levels than those with cervical injuries, these differences did not reach statistical significance in the pooled analyses. Similarly, injury severity and aetiology were not significantly associated with reintegration outcomes. These findings contrast with those of several international studies that have reported stronger associations between injury characteristics and participation outcomes (Craig et al. 2015; Reinhardt et al. 2016). One possible explanation is that environmental, social and economic barriers may exert a stronger influence on reintegration than biological impairment in low-resource settings, thereby attenuating the observable effects of injury-related factors (Atobatele et al. 2018; Buys et al. 2022; Serres-Lafontaine et al. 2023). Biological factors, such as injury level, severity, degree of motor and sensory impairment and the presence of secondary complications (e.g. spasticity or pain), may exert a comparatively weaker influence on reintegration outcomes (Atobatele et al. 2018).

The triangulation of individual and pooled analyses strengthened the confidence in the observed patterns, particularly for age and employment status. However, the exploratory nature of our study, reliance on secondary data and absence of multivariable adjustment limit causal inferences. Unmeasured factors, such as time since injury, environmental accessibility, transport availability and social policy support, may confound the observed associations and warrant further investigation.

Overall, the findings underscore the importance of considering the sociodemographic context and injury characteristics when planning rehabilitation and community-based interventions. In settings such as South Africa, where structural and socioeconomic constraints are pronounced, interventions aimed at improving employment opportunities, social inclusion and environmental accessibility may be as critical as impairment-focused rehabilitation in supporting successful community reintegration.

### Clinical implications

Understanding the sociodemographic and injury factors associated with community integration can help therapists to identify PWSCIs at greater risk of poor community reintegration and provide more holistic and individualised rehabilitation programmes to support more successful reintegration into the community. Specifically, employment status, injury mechanism and injury completeness may guide clinicians in identifying patients at risk of poor community reintegration and informing tailored interventions. Additionally, our study may inform the development of risk screening tools to indicate when PWSCIs during rehabilitation need to be flagged to receive more intensive community reintegration support in the future.

### Strengths and limitations

A key strength of our study is the secondary analysis of two independent South African datasets that examined community reintegration among individuals with SCI using the same outcome measure: the Reintegration to Normal Living Index. The pooled analysis increased the overall sample size to 182 participants, thereby improving the statistical power compared with that of the individual primary studies. In addition, the use of triangulation, whereby findings from individual datasets were compared with pooled results, strengthened the confidence in associations that were consistent across the analyses.

Despite these strengths, several limitations should be acknowledged in our study. Of the original records available across both datasets, only those with complete data on sociodemographic variables, injury characteristics and RNLI scores were included in the final analysis. Consequently, records with missing data were excluded, and a complete case analysis was performed. Although this approach ensured analytical consistency, it may have introduced selection bias if the excluded participants differed systematically from those who were included.

The use of non-probability convenience sampling in both primary studies further limits representativeness and may restrict the generalisability of the findings to the broader population of people with SCI in South Africa. In addition, although predefined homogeneity criteria were applied, the datasets differed in terms of sample size, sex distribution, employment status and injury-level composition. This residual heterogeneity may have influenced the pooled estimates, despite efforts to minimise bias through variable harmonisation and triangulation.

The cross-sectional design of both primary studies precludes causal inferences. The observed associations between sociodemographic factors, injury characteristics and community reintegration reflect correlations at a single point in time and cannot establish temporal or causal relationships. Furthermore, the secondary nature of the data limited the availability of potentially important confounders, such as time since injury, environmental accessibility, transport availability and policy or social support factors. Therefore, multivariate adjustment and data weighting were not feasible, and residual confounding could not be excluded.

These limitations indicate that the findings should be interpreted as exploratory and hypothesis-generating only. Future longitudinal studies using representative sampling and comprehensive measurements of contextual factors are needed to clarify the causal pathways influencing community reintegration among individuals with SCI.

## Conclusion

In this secondary analysis of two South African datasets, community reintegration among individuals with SCI was associated with sociodemographic factors, particularly age, sex and employment status. Injury-related characteristics showed weak and nonsignificant associations. These findings underscore the relevance of social and contextual factors in understanding reintegration outcomes and highlight the need for longitudinal, context-sensitive research to inform rehabilitation and community support strategies.

## References

[CIT0001] Akter, F., Islam, S., Haque, O., Hossain, A., Hossain, K., Imran, M. et al., 2019, ‘Barriers for individuals with spinal cord injury during community reintegration: A qualitative study’, *International Physical Medicine & Rehabilitation Journal* 7, 2.

[CIT0002] Atobatele, K.O., Olaleye, O.A., Fatoye, F.A. & Hamzat, T.K., 2018, ‘Relationships between community reintegration and clinical and psychosocial attributes in individuals with spinal cord injury in a Nigerian city’, *Topics in Spinal Cord Injury Rehabilitation* 24, 306–314. 10.1310/sci16-00055 30459493 PMC6241226

[CIT0003] Barclay, L., Robins, L., Migliorini, C. & Lalor, A., 2021, ‘Community integration programs and interventions for people with spinal cord injury: A scoping review’, *Disability and Rehabilitation* 43, 3845–3855. 10.1080/09638288.2020.1749889 32356499

[CIT0004] Buys, E., Nadasan, T., Pefile, N., Ogunlana, M.O. & Naidoo, D., 2022, ‘Clinical and sociodemographic determinants of community reintegration in people with spinal cord injury in eThekwini Municipality, KwaZulu-Natal province’, *South African Journal of Physiotherapy* 78, 1631. 10.4102/sajp.v78i1.1631 35747514 PMC9210146

[CIT0005] Carr, J., Kendall, M., Amsters, D., Pershouse, K., Kuipers, P., Buettner, P. et al., 2017, ‘Community participation of individuals with spinal cord injury living in Queensland, Australia’, *Spinal Cord* 55, 192–197. 10.1038/sc.2016.169 27897188

[CIT0006] Craig, A., Nicholson Perry, K., Guest, R., Tran, Y. & Middleton, J., 2015, ‘Adjustment following chronic spinal cord injury: Determining factors that contribute to social participation’, *British Journal of Health Psychology* 20, 807–823. 10.1111/bjhp.12143 26037456

[CIT0007] Dwyer, K.J. & Mulligan, H., 2015, ‘Community reintegration following spinal cord injury: Insights for health professionals in community rehabilitation services in New Zealand’, *New Zealand Journal of Physiotherapy* 43, 75–85. 10.15619/NZJP/43.3.02

[CIT0008] Grimes, D.A. & Schulz, K.F., 2002, ‘Bias and causal associations in observational research’, *Lancet* 359, 248–252. 10.1016/S0140-6736(02)07451-2 11812579

[CIT0009] Gupta, S., Jaiswal, A., Norman, K. & Depaul, V., 2019, ‘Heterogeneity and its impact on rehabilitation outcomes and interventions for community reintegration in people with spinal cord injuries: An integrative review’, *Topics in Spinal Cord Injury Rehabilitation* 25, 164–185. 10.1310/sci2502-164 31068748 PMC6496968

[CIT0010] Kanyoni, M., Wikmar, L.N., Philips, J., Joseph, C. & Tumusiime, D.K., 2024, ‘Incidence and etiology of traumatic spinal cord injury in Rwanda: A prospective population-based study’, *Frontiers in Neurology* 15, 1373893. 10.3389/fneur.2024.1373893 39233676 PMC11371736

[CIT0011] Lijodi, B., Titus, A., Louw, Q. & Joseph, C., 2025, ‘Activity limitations, participation restrictions, and environmental barriers among persons with traumatic spinal cord injury in Kenya’, *Disability and Rehabilitation* 47, 1156–1162. 10.1080/09638288.2024.2365413 38950895

[CIT0012] Liu, Y., Yang, X., He, Z., Li, J., Li, Y., Wu, Y. et al., 2023, ‘Spinal cord injury: Global burden from 1990 to 2019 and projections up to 2030 using Bayesian age-period-cohort analysis’, *Frontiers in Neurology* 14, 1304153. 10.3389/fneur.2023.1304153 38116113 PMC10729761

[CIT0013] Mashola, K.M., 2022, ‘The presence of pain and its impact on functioning and disability in manual wheelchair users with spinal cord injury: A framework for self-management’, PhD thesis, University of Pretoria, Pretoria.

[CIT0014] Mashola, M. & Mothabeng, D., 2019, ‘Associations between health behavior, secondary health conditions, and quality of life in people with spinal cord injury’, *African Journal of Disability* 8, 463. 10.4102/ajod.v8i0.463 31309047 PMC6620481

[CIT0015] Morwane, R., Dada, S. & Bornman, J., 2021, ‘Barriers to and facilitators of employment of persons with disabilities in low- and middle-income countries: A scoping review’, *African Journal of Disability*, 10, 833. 10.4102/ajod.v10i0.833 34230882 PMC8252133

[CIT0016] Nizeyimana, E., Phillips, & Joseph, C., 2024, ‘Psychosocial reintegration following traumatic spinal cord injury in South Africa: The influence of employment, injury characteristics, and living situation’, *Journal of Spinal Cord Medicine* 47, 255–262. 10.1080/10790268.2021.2016306 35007494 PMC10885739

[CIT0017] Nweke, M., Van Vuuren, M., Bester, K., Maritz, A., Van Vuuren, L., Vilakazi, Y. et al., 2025, Theoretical considerations and data triangulation in a secondary analysis of exposure-outcome association: A case protocol.

[CIT0018] Pazzi, C., Farrehi, C., Capron, M., Anderson, K., Richardson, B. & Stillman, M., 2021, ‘An assessment of which sociodemographic and spinal cord injury-specific characteristics influence engagement with experimental therapies and participation in clinical trials’, *Top Spinal Cord Injury Rehabilitation* 27, 28–39. 10.46292/sci20-00070 PMC8604506 34866886

[CIT0019] Quadri, S.A., Farooqui, M., Ikram, A., Zafar, A., Khan, M.A., Suriya, S.S. et al., 2020, ‘Recent updates on the basic mechanisms of spinal cord injury’, *Neurosurgical Review* 43, 425–441. 10.1007/s10143-018-1008-3 29998371

[CIT0020] Reinhardt, J.D., Ballert, C., Brinkhof, M.W. & Post, M.W., 2016, ‘Perceived impact of environmental barriers on participation among people living with spinal cord injury in Switzerland’, *Journal of Rehabilitation Medicine* 48, 210–218. 10.2340/16501977-2048 26926923

[CIT0021] Seabold, S. & Perktold, J., 2010, ‘Statsmodels: Econometric and statistical modelling with Python’, in Proceedings of the 9th Python in Science Conference (SciPy 2010), pp. 92–96, Austin, TX.

[CIT0022] Serres-Lafontaine, A.D., Labbé, D., Batcho, C.S., Norris, L. & Best, K.L., 2023, ‘Social participation of individuals with spinal injury using wheelchairs in rural Tanzania after peer and entrepreneurial skills training’, *African Journal of Disability* 12, 975. 10.4102/ajod.v12i0.975 36756462 PMC9900306

[CIT0023] Swart, E., Bitzer, E.M., Gothe, H., Harling, M., Hoffmann, F., Horenkamp-Sonntag, D., et al., 2016, ‘A consensus German reporting standard for secondary data analyses, version 2 (STROSA-STandardisierte BerichtsROutine für SekundärdatenAnalysen)’, *Gesundheitswesen* 78(S 01), e145–e160. 27351686 10.1055/s-0042-108647

[CIT0024] Van der Westhuizen, L., 2016, ‘Correlation between physical activity and community participation in individuals with spinal cord injury’, MPhysT dissertation, University of Pretoria.

[CIT0025] Van der Westhuizen, L., Mothabeng, D.J. & Nkwenika, T.M., 2017, ‘The relationship between physical fitness and community participation in individuals with spinal cord injury’, *South African Journal of Physiotherapy* 73, 354. 10.4102/sajp.v73i1.354 30135904 PMC6093096

[CIT0026] Wickham, R.J., 2019, ‘Secondary analysis of research data’, *Journal of the Advanced Practitioner in Oncology* 10, 395. 10.6004/jadpro.2019.10.4.7 33343987 PMC7520737

